# Faster key-press responses to front vowels than back vowels when matching heard vowels with represented vowels

**DOI:** 10.3758/s13423-026-02935-2

**Published:** 2026-06-22

**Authors:** Toshimune Kambara, Masahiro Shinya, Mizuki Yoshio, Konatsu Ishimizu, Shihoko Kakuno, Tomoki Nakatani, Moe Otsuka, Ami Sunagawa

**Affiliations:** 1 Graduate School of Humanities and Social Sciences, Hiroshima University, Higashi-Hiroshima City, 1-1-1 Kagamiyama, Hiroshima, 739-8524 Japan; 2 School of Education, Hiroshima University, Hiroshima, Japan

**Keywords:** Japanese, Vowels, Key-press responses, Response time, Vowel-motor symbolism

## Abstract

This study investigated key-press responses to five spoken Japanese vowels: [ɑ], [i], [ɯ], [e], and [o]. In the first experiment, 30 participants pushed only a single key when they listened to spoken Japanese vowels (response task). In the second experiment, another 29 participants matched the heard vowels with represented vowels by pushing one of five corresponding keys (matching task). The results of the first experiment showed no difference in the response times to the five vowels. In the second experiment, the response times to [i] and [e] were faster than those to [ɯ] and [o]. These results suggest that front vowels promote faster key-press responses than back vowels when the keys corresponding to the heard vowels are consciously selected.

Linguistic features (e.g., spoken sounds, written forms, braille, finger signs) are connected to referential features, including sensory-motor or emotional attributes (Paivio, 1986, 2007). The linguistic and referential relationships are both arbitrary (de Saussure, 2011) and nonarbitrary (symbolic; Sapir, 1929). The nonarbitrariness of the relationship between language and referents may be influenced by the structure of the human body (specifically, the vocal tract). This view is supported by the fact that only four patterns of word sounds emerge at the early developmental stages of language (MacNeilage & Davis, 2000). Cross-modal correspondence is a fundamental cognitive function, where information from different sensory modalities is integrated (Spence, 2011). In the domain of language, this is exemplified by symbolism, where linguistic features cross-modally correspond to specific sensorimotor features (Crisinel et al., 2012; Pathak et al., 2020). For instance, words involving long vowels are connected to sweet imagery compared with those involving short vowels (Pathak et al., 2020). Such phenomena have been investigated; previous studies showed that nonlinguistic features are symbolically connected to written (Cuskley et al., 2017; Hayashi et al., in press) and spoken linguistic forms (Lockwood & Dingemanse, 2015; Ohala, 1994; Ramachandran & Hubbard, 2001). Additionally, such symbolism between linguistic and referential information can also promote word learning in native and second languages (Imai & Kita, 2014) and body action (Vainio & Vainio, 2021).

Linguistic information can also be nonarbitrarily connected to emotional information. The first phoneme in a word can predict emotional valence (Adelman et al., 2018). People prefer words that involve front-to-back mouth movements (Ingendahl et al., 2022) or consonants articulated at the front of the mouth (Maschmann et al., 2020). Such linguistic and emotional connections have been shown for phonemes or graphemes. In a letter search task, reactions were faster for graphemes with high arousal (Schmidtke & Conrad, 2018). Previous research showed that /i/ was used for positive responses, while /u/ and /o/ were not, in both German and Japanese (Körner & Rummer, 2023). Other studies also support that words including /i/ or /i:/ are associated with positive valence more than those including /u/ and /o/ or /o:/ (Garrido & Godinho, 2021; Rummer & Schweppe, 2019; Rummer et al., 2014). Since the similarity among positive stimuli is higher than that among negative stimuli, this emotional similarity boosts processing speed (Unkelbach et al., 2008). If response speed to a stimulus connects to its emotional valence, then the response speed to a positive linguistic stimulus should be faster than the response speed to a negative linguistic stimulus. As mentioned above, because front vowels (e.g., /i/) are more positive than back vowels (/o/), the response speed to front vowels is expected to be faster. In this case, emotional information can mediate the connections between linguistic and sensorimotor information (emotion mediation hypothesis; see Palmer et al., 2013, for music-color associations).

One unique linguistic and cognitive feature of the Japanese language is the use of *kakegoe*—vocalizations that accompany physical exertion. For example, when standing up, Japanese speakers often say “*yoisho*,” “*iyoisho*,” or “*yokkoisho*.” Previous research suggests that such vocalization can facilitate physical actions in native Japanese speakers. (In Sato et al., 2013, the throwing task used a free-vowel condition rather than the frontvowels [i] and [e].) facilitated physical performance (e.g., grip strength, vertical jump height) compared with a nonvocalization condition. However, no research has examined motor response speeds when Japanese speakers merely hear these spoken vowels.

The aim of this study was to identify the differences among key-press responses to five spoken Japanese vowels ([ɑ], [i], [ɯ], [e], and [o]). Key-press responses were selected as the simplest motor responses to spoken vowels. Based on Labrune (2012) and other studies (see Kawahara et al., 2017; Okada, 1999; Vance, 2008, for a review of Japanese vowels), we used the phonetic realizations [ɑ], [i], [ɯ], [e], and [o] for the phonemes /a/, /i/, /u/, /e/, and /o/, respectively. The vowels [i] and [e] are categorized as front vowels, whereas [ɯ] and [o] are categorized as back vowels (Labrune, 2012; Okada, 1999; Vance, 2008). Meanwhile, [ɑ] is categorized as a central (Labrune, 2012; Okada, 1999) or back (Vance, 2008) vowel. To investigate sound-symbolic key-press responses to these five Japanese spoken vowels, we conducted two experiments. In Experiment 1, the participants immediately pressed a key when they listened to a spoken vowel (response task). In Experiment 2, other participants selected and pressed an appropriate key from among the five pseudorandomly ordered keys when they listened to a spoken vowel (matching task). In the matching task, the participants had to correctly determine which vowels they had heard.

We made two predictions. Experiment 1 was designed as a baseline control to ensure that the acoustic properties of the different vowels do not inherently cause differences in simple motor execution speeds. In Experiment 1, we predicted that no significant difference would occur in the response times to vowels because the participants simply responded to the onset of heard sounds by using a single key without any cognitive matching process. Establishing this baseline is essential to confidently attribute any differences found in the subsequent experiment to the cognitive processes of vowel–motor mapping. In Experiment 2, we predicted that the responses to front vowels (i.e., [i] and [e]) would be faster than those to back vowels (i.e., [ɯ] and [o]) because the key responses to front vowels would be more facilitated by the positive emotions to the front vowels than those to the back vowels when matching the heard vowels with the vowels previously registered in their long-term memories.

## Experiment 1

### Methods

#### Participants

Thirty healthy native Japanese speakers (seven women, 22 men, and one person who identified as other; mean age = 41.27 years, *SD* = 8.55 years, age range: 26–61 years) participated in this experiment. This sample size provided adequate power because the total number of trials for each condition is more than 1,600 trials (Brysbaert & Stevens, 2018). They were recruited from Crowdworks, Inc., which is a Japanese crowdsourcing platform. Before the experiment, participants read an explanation of the study’s objectives and ethical considerations. If they agreed to participate, they pressed a key to indicate their informed consent. After the experiment, participants received 143 Japanese yen as remuneration, inclusive of the miscellaneous fees from Crowdworks, Inc. This study was approved by the Ethics Committee of the Graduate School of Humanities and Social Sciences at Hiroshima University.

#### Stimuli

Five Japanese vowels ([ɑ], [i], [ɯ], [e], and [o]) obtained from four native Japanese speakers (three women and one man, who were the fourth, fifth, sixth, and eighth authors) were recorded as the stimuli used in Experiments 1 and 2. The total number of different auditory stimuli was 20 (5 vowels × 4 speakers). The stimuli were recorded using an IC recorder (Sony ICD-SX1000) in a silent room at the authors’ institution. After recording the spoken vowels, we normalized them using Audacity (an open-source sound editing software; https://www.audacityteam.org/). To analyze the mean f0 and intensity of the vowels, we utilized Praat (Boersma & Weenink, 2025) and Praat scripts (https://github.com/Syuparn/praat_batch_analysis), after converting the original MP3 files to WAV files using Sound Converter. The mean f0 values of [ɑ], [i], [ɯ], [e], and [o] were 181 Hz, 193 Hz, 202 Hz, 201 Hz, and 241 Hz, respectively. The mean intensities were 73.91 dB, 73.55 dB, 75.51 dB, 75.73 dB, and 75.38 dB, respectively. Finally, to measure vowel duration, we used Praat and a specific script (https://gist.github.com/Yiling-Huo/2964a50ab5fcdf78a6544d5da29e83d2), confirming that all auditory stimuli had an identical duration of 327.62 ms.

#### Experimental procedures

The experiment was conducted using the Gorilla Experiment Builder (https://gorilla.sc/) (Anwyl-Irvine et al., 2020). In the response task, when the participants listened to each spoken vowel, they immediately pressed the space bar on a standard computer keyboard. Hand position and finger assignment were not restricted. The task consisted of 300 trials in total. Each of the five vowels was presented 60 times (4 speakers × 15 repetitions per vowel) in a fully randomized order. To prevent participants from predicting stimulus onset, a fixation cross ( +) was presented between trials for a randomized duration of 500 ms, 1,000 ms, or 1,500 ms. The background of the screen was white, whereas the fixations and instructions at the beginning and end were written in black. Before the experiment, the participants performed five practice trials. These trials were excluded from the analyses. The experiment took approximately 10 min to complete.

#### Analyses

We performed linear mixed-effects modeling to compare the response times to vowels. The dependent variables were the response times to vowels, and the independent variables (fixed effects) were the five vowels ([ɑ], [i], [ɯ], [e], and [o]). Random effects included participants and stimuli as random intercepts (Kambara & Umemura, 2021); random slopes were not fitted. Therefore, the R syntax used was lmer (RT ~ condition + (1 | ID) + (1 | stimuli), data = data). Finally, a Type III analysis of variance (ANOVA) with Kenward–Roger approximation was conducted using the linear mixed-effects model. We applied a two-step outlier exclusion procedure: (1) response times shorter than 150 ms or longer than 2,000 ms were removed, and (2) data points falling outside the mean ± 2 standard deviations were excluded. The mean and standard deviations of the response times were calculated by the *Rmisc* package (Hope, 2022) on R (R Core Team, 2024). Other R packages used included *afex* (Singmann et al., 2025), *dplyr* (Wickham et al., 2023), *effectsize* (Ben-Shachar et al., 2020), *emmeans* (Lenth, 2025), *lmerTest* (Kuznetsova et al., 2017), *performance* (Lüdecke et al., 2021), and *writexl* (Ooms, 2024). The data and scripts used in this study are available on the Open Science Framework (Kambara & Yoshio, 2026).

### Results

For the dataset with cutoffs, Table 1 shows the means and standard deviations of response times to vowels. In Experiment 1, the rejection rates after cutoffs for [ɑ], [i], [ɯ], [e], and [o] were 4.50% (81/1,800), 5.83% (105/1,800), 4.89% (88/1,800), 5.22% (94/1,800), and 6.28% (113/1,800), respectively. A Type III ANOVA with Kenward–Roger approximation revealed no significant main effect of vowels on response times in the linear mixed-effects model, *F*(4,15.00) = 0.41, *p* = 0.80, η_p_^2^ = 0.10. Regarding model fit, the conditional and marginal *R*^2^ values were 0.43 and 0.0007, respectively.

**Table 1:** Mean and standard deviations of response times in Experiment 1

Vowels	*M*	*SD*
[ɑ]	417.46	116.12
[e]	420.60	120.31
[i]	422.78	118.60
[o]	423.73	121.25
[ɯ]	425.68	118.74

Rmisc (Hope, 2022) and R (R Core Team, 2024) were used

*M *mean, *SD *standard deviation

[] represents International Phonetic Alphabet (IPA)

### Discussion

In Experiment 1, we did not find any differences in the key-press response times to the five Japanese spoken vowels. Because the participants simply pressed a key in response to the onset of any vowel without needing to consciously identify or match the specific sound, no difference in response times was expected. However, if participants are required to consciously identify and match the heard vowels, their responses to front vowels should be faster due to the emotional positivity associated with these stimuli (Unkelbach et al., 2008). Therefore, in Experiment 2, we examined key-press responses to vowels when participants selected matched vowels from heard vowels.

## Experiment 2

### Methods

#### Participants

Twenty-nine healthy native Japanese speakers participated in this experiment (13 women and 16 men; mean age = 40.72 years; *SD* = 9.85 years; age range: 23–60 years). This sample size provided adequate power because the total number of trials for each condition is more than 1,600 trials (Brysbaert & Stevens, 2018). The participants were not the same as those who had performed the response task in Experiment 1. The recruitment procedures, ethical approval, and informed consent process were identical to those in Experiment 1.

#### Stimuli

The stimuli were the same 20 recorded Japanese vowels used in Experiment 1.

#### Experimental procedures

Experiment 2 was also conducted using the Gorilla Experiment Builder (https://gorilla.sc/) that was used in Experiment 1 (Anwyl-Irvine et al., 2020). In the matching task, when the participants listened to each spoken vowel, they immediately selected and pressed the corresponding key by using a right index finger. Finger placement was not fixed. Each vowel was assigned to a specific key on a standard computer keyboard (i.e., no external response device was used). Since the experiment was conducted online using participants’ own devices, the physical response keys were not visually marked. Instead, the specific key-to-vowel mapping was visually presented on the screen. Participants were instructed to press the response keys (keys 1 to 5, arranged from left to right) according to the button array displayed on their screen. To counterbalance the spatial arrangement of the vowels, the key-to-vowel mapping followed one of the five patterns summarized in Table 2, which were randomized between trials. Each key-order pattern was presented three times for the recordings of each of the five vowels by the four native Japanese speakers (3 repetitions × 5 patterns × 4 speakers × 5 vowels = 300 total trials in a fully randomized order). To prevent participants from predicting stimulus onset, a fixation cross was presented between trials for a randomized duration of 500 ms, 1,000 ms, or 1,500 ms. The background of the screen was white, whereas fixations and instructions were written in black. Before the experiment, the participants performed five practice trials. These trials were not included in the analyses. Participants performed this experiment in approximately 10 min.

**Table 2:** Patterns of the key order are shown on the screen in Experiment 2

	Key 1	Key 2	Key 3	Key 4	Key 5
Pattern 1	[ɑ]	[i]	[ɯ]	[e]	[o]
Pattern 2	[i]	[ɯ]	[e]	[o]	[ɑ]
Pattern 3	[ɯ]	[e]	[o]	[ɑ]	[i]
Pattern 4	[e]	[o]	[ɑ]	[i]	[ɯ]
Pattern 5	[o]	[ɑ]	[i]	[ɯ]	[e]

The keys from Key 1 to Key 5 were ordered from left to right

#### Analyses

First, we conducted generalized linear mixed-effects modeling to specify the differences between the accuracies of the five vowels. We used all trials for the analyses. The dependent variables were the responses to the vowels (0: incorrect; 1: correct), and the independent variables (fixed effects) were the five vowels ([ɑ], [i], [ɯ], [e], and [o]). The random effects included participants and items as random intercepts (Kambara & Umemura, 2021). We used glmer (performance ~ condition + (1 | ID) + (1 | stimuli), data = data, family = “binomial”) as the R syntax. Finally, we tested the significance of the fixed effect using a likelihood ratio test based on the generalized linear mixed-effects model.

Second, we conducted linear mixed-effects analyses to compare the response times with vowels. We used only correct trials for the analyses. Additionally, we applied a two-step outlier removal procedure for the response time analyses using correct trials. First, we excluded response times shorter than 150 ms or longer than 2,000 ms. Second, we excluded response times falling outside the mean ± 2 standard deviations. The dependent variables were the response times, and the independent variables (fixed effects) were the five vowels ([ɑ], [i], [ɯ], [e], and [o]). Random effects comprised participants and stimuli as the random intercepts only. Random slopes were also not fitted. Therefore, the R syntax used was lmer (RT ~ condition + (1 | ID) + (1 | stimuli), data = data). Finally, we conducted a Type III ANOVA with Kenward–Roger approximation using the linear mixed-effects model.

Statistical analyses were conducted using R (R Core Team, 2024) with the same set of packages as in Experiment 1. We used all trials for the descriptive statistics of accuracy rates, whereas we used correct trials excluding outliers for the descriptive statistics of response times. The data and scripts used in this experiment are available on the Open Science Framework (Kambara & Yoshio, 2026).

### Results

The mean and standard deviations of the accuracy rates, as well as the response times calculated from the correct trials retained after the cutoffs, are listed in Table 3. In Experiment 2, the outlier rejection rates out of the correct trials for [ɑ], [i], [ɯ], [e], and [o] were 12.87% (220/1,709), 8.53% (144/1,688), 13.72% (234/1,705), 9.62% (165/1,715), and 17.91% (303/1,692), respectively.

**Table 3:** Mean and standard deviations of accuracies and response times in Experiment 2

Vowels	Accuracies		Response times of correct trials	
	*M*	*SD*	*M*	*SD*
[ɑ]	0.98	0.13	1,184.78	300.06
[e]	0.99	0.12	1,167.71	293.02
[i]	0.97	0.17	1,092.18	289.99
[o]	0.97	0.16	1,239.70	289.28
[ɯ]	0.98	0.14	1,211.43	306.66

Rmisc (Hope, 2022) and R (R Core Team, 2024) were used

*M* mean, *SD *standard deviation

[] represents International Phonetic Alphabet (IPA)

There were significant differences in the response times to vowels, *F*(4,15.02) = 51.69, *p* < 0.001, η_p_^2^ = 0.93, Conditional* R*^2^ = 0.38, Marginal *R*^2^ = 0.03. However, there were no significant differences in the accuracy of vowels, χ^2^(4) = 1.49, *p* = 0.83, Conditional *R*^2^ = 0.22, Marginal *R*^2^ = 0.009. As shown in Table 4, the response times to the vowel [i] were faster than those to the vowels [ɑ], [ɯ], [e], and [o]. The response times to the vowel [e] were faster than those to the vowels [ɯ] and [o] but not those to the vowel [ɑ]. The response times to the vowel [ɑ] were faster than those to the vowel [o] but not those to the vowel [ɯ]. Finally, the response times to the vowel [ɯ] were faster than those to the vowel [o]. These findings suggest that the response times to front vowels, including [i] and [e], were significantly faster than response times to back vowels, including [ɯ] and [o].

**Table 4:** Contrasts between response times to vowels in Experiment 2

Contrasts	*Estimate*	*SE*	*t* ratio	95% CI LL	UL	*p*	*d*
[ɑ] vs. [e]	23.2	12.1	1.913	− 14.350	60.738	0.3530	0.0917
[ɑ] vs. [i]	96.6	12.1	7.965	59.056	134.166	< .0001	0.3819
[ɑ] vs. [o]	− 68.6	12.3	− 5.568	− 106.473	− 30.762	0.0004	− 0.2712
[ɑ] vs. [ɯ]	− 30.5	12.2	− 2.495	− 68.166	7.207	0.1437	− 0.1205
[e] vs. [i]	73.4	12.1	6.089	35.966	110.868	0.0002	0.2902
[e] vs. [o]	− 91.8	12.3	− 7.489	− 129.568	− 54.056	< .0001	− 0.3629
[e] vs. [ɯ]	− 53.7	12.1	− 4.418	− 91.256	− 16.091	0.0040	− 0.2121
[i] vs. [o]	− 165.2	12.3	− 13.474	− 202.990	− 127.468	< .0001	− 0.6531
[i] vs. [ɯ]	− 127.1	12.2	− 10.459	− 164.680	− 89.500	< .0001	− 0.5023
[o] vs. [ɯ]	38.1	12.3	3.090	0.254	76.024	0.0481	0.1507

For the contrasts between response times to vowels, we applied Tukey’s method in the *emmeans* package (Lenth, 2025) and R (R Core Team, 2024)

*SE *standard error, *CI *confidence interval, *LL *lower limit, *UL *upper limit, *d *Cohen’s d

## General discussion

To verify whether spoken front vowels in Japanese facilitate key-press responses, we conducted two behavioral experiments (Experiments 1 and 2). These experiments were designed to distinguish between responses to simply heard sounds (Experiment 1) and responses associated with heard sounds (Experiment 2). In the response task of Experiment 1, participants simply pressed a key when they heard each spoken Japanese vowel. As predicted, no significant difference in the response times to vowels was observed in Experiment 1. In the matching task of Experiment 2, other participants who did not perform the response task of Experiment 1 selected a pseudorandomly presented key corresponding to each spoken vowel. The results of Experiment 2 showed that the response times to the front vowels [i] and [e] were significantly faster than those to the back vowels [ɯ] and [o]. The results of both experiments showed that the auditory perception of front vowels could symbolically boost the key-press responses to spoken vowels when people consciously matched heard vowels with represented vowels. The findings and limitations of this study are discussed in the following paragraphs.

In Experiment 1, we did not find significant differences in the key-press response times to vowels. This may have occurred because the participants might not have matched each detected vowel with each represented vowel in their long-term memories. In the response task of Experiment 1, the participants immediately pushed a key when they detected any auditorily presented vowel. In that case, the participants might not have needed to recognize the vowel they detected. Therefore, each detected vowel may not have facilitated the response time of the key-press response if the participants did not match each detected vowel with each represented vowel.

Meanwhile, in the matching task of Experiment 2, the responses to front vowels ([i] and [e]) were faster than those to back vowels ([ɯ] and [o]). The results supported the second prediction based on previous findings associated with the emotion mediation hypothesis—that emotional information mediates the associations between linguistic and sensorimotor information. Previous research has reported that front vowels (e.g., /i/ or /i:/) are associated with more positive valence than back vowels (e.g., /u/ or /u:/; Garrido & Godinho, 2021; Körner & Rummer, 2023; Rummer & Schweppe, 2019; Rummer et al., 2014). Since the positivity of stimuli can promote processing speed (Unkelbach et al., 2008), these findings suggest that the emotional information of vowels may affect key-press speeds.

Previous studies have shown that front vowels facilitate the response speed of a precision grip using the thumb and index finger, compared to back vowels (Hajj et al., 2023; Tiainen et al., 2017; Vainio et al., 2013, 2014). For example, Vainio et al. (2014) showed that reading or hearing syllables involving front vowels (e.g., [i]) boosts the response speed of a precision grip using the thumb and index finger, compared with back vowels (e.g., [ɑ]). When pronouncing vowels, the tongue position comprises two dimensions (Vance, 2008). In the vertical dimension of the tongue position, when the highest position of the tongue is far from the roof of the mouth, low vowels are produced. When the highest position of the tongue is close to the roof of the mouth, high vowels are produced (Vance, 2008). In the horizontal dimension of the tongue position, when pronouncing front vowels, the highest point of the tongue is at the front, whereas the highest point of the tongue is at the back when pronouncing back vowels (Vance, 2008). In Experiment 2, the rank order of the response speed was [i], [e], [ɑ], [ɯ], and [o]. Interestingly, the rank order of the response speed almost corresponds to the horizontal order of tongue position from front to back when pronouncing vowels (Okada, 1999). However, the horizontal order of [ɑ], [ɯ], and [o] remains a controversial subject among researchers (e.g., Labrune, 2012; Okada, 1999; Vance, 2008). Additionally, vowel frequency does not appear to affect key-press responses. Although previous research indicates that the frequency of /e/ is lower than that of other vowels in Japanese (Tamaoka & Makioka, 2004), responses to front vowels (including [i] and [e]) were faster than those to back vowels (including [ɯ] and [o]). Therefore, the frequency effect on vowels may not appear in this study. For both Japanese and German speakers, names containing the front vowel /i/ are associated with positive facial valence, whereas names containing the back vowels /o/ or /u/ are not (Körner & Rummer, 2023). Furthermore, people with a positive mood generated words including /i:/, while people with a negative mood generated words including /o:/ (Rummer et al., 2014).

In Experiment 2, accuracy rates showed no significant differences across responses to the vowel stimuli. The task, which required participants to select a key associated with each presented vowel using their right index finger, was straightforward. Vainio et al. (2013) reported that precision grip actions using the thumb and index finger involve fewer errors than power grip actions using the little, ring, and middle fingers. Consistent with the advantage of precision grip actions, our findings suggest that the motor responses utilizing the index finger are less demanding for participants. As the mean error rates for Experiment 2 remained below 3%, it can be concluded that responding to auditory stimuli with the right index finger was so simple that performance errors were minimal.

The interaction between vowel perception and motor responses observed in the present study may be salient within the Japanese language for several linguistic and cultural reasons. First, as noted in the Introduction, Japanese culture utilizes *kakegoe* (effort vocalizations, such as “*yoisho*”), a practice that establishes a link between linguistic sounds and physical motion. Second, the Japanese language possesses a rich lexical stratum of sound-symbolic words (mimetics), wherein vowels systematically encode sensory-motor and emotional information (e.g., Imai & Kita, 2014). This linguistic environment likely heightens native speakers’ sensitivity to the embodied associations of speech sounds. Finally, because Japanese features a simple five-vowel system within a mora-timed, consonant–vowel (CV) syllable structure (Vance, 2008), vowels are prominent in most utterances. Thus, the emotional and motoric affordances of vowels might be more activated and observable in Japanese speakers compared to speakers of languages with more complex vowel systems.

In the present study, we verified the way that humans associate the actions of body parts with linguistic forms. Regarding linguistic and sensorimotor connections, humans connect linguistic forms with visual (Obinna et al., 2025), auditory (Cosper et al., 2020), olfactory (González et al., 2006), gustatory (Barrós-Loscertales et al., 2012; Wang et al., 2025), and motor (Hauk et al., 2004) referents, even when such connections are arbitrary. Furthermore, sound symbolism promotes the learning of verbs (Imai et al., 2008). 

While the present study suggests results supporting the emotion mediation hypothesis (i.e., that emotional information can mediate the connections between linguistic and sensorimotor information)—specifically, that emotional meaning associated with linguistic sounds shortens response times—this effect is thought to exist not only at the phonemic level but also at more complex linguistic levels. For example, focusing on structural features rather than at the phonemic or lexical level, poetic meter (e.g., regulating the number of morae per line) and alliteration (e.g., repeating the initial consonant of lines) have been shown to influence the emotional meaning of poetry (Yoshio & Kambara, 2025).

This study includes six limitations and future directions. First, in this study, we only examined native Japanese speakers’ key-press responses to Japanese spoken vowels. However, as a direction for future research, studies could investigate whether key-press responses to front vowels are universally faster than those to back vowels in any foreign language.

Second, this study has several limitations regarding the experimental methodology. Although we did not examine the relationship between vowel responses and key size, a previous study reported that key size affects these responses (Heurley et al., 2023). Specifically, they found that front vowels (e.g., reading “TI”) facilitated pressing small keys, whereas back vowels (e.g., reading “KA”) facilitated pressing large keys. Future studies should examine the interaction between vowel response times and key size across different languages, specifically from the perspective of vowel-size symbolism.

Third, experimental control over motor execution was limited; finger assignment, starting position, and hand usage were not strictly standardized. Furthermore, participants used standard computer keyboards rather than specialized response devices. These methodological factors may have influenced the results in both experiments.

Fourth, although this study only focused on key-press responses to heard vowels, other body actions are affected by vowels. Vainio et al. (2019) showed that leg forward actions from back to front are linked to front vowels, and leg backward actions from front to back are linked to back vowels.

Fifth, a limitation of the present study is related to the nature of the experimental task, which inherently involved a visual search component. Because participants were required to visually locate the key assigned to a specific vowel on the screen, variations in the time required to visually process and find different vowels (e.g., [i] versus [o]) might have influenced the response times. Although we minimized this confounding factor by counterbalancing the key-to-vowel assignments across participants, we cannot entirely rule out the possibility that the cognitive load associated with this visual search added noise to the response-time data.

Sixth, a limitation of the present study concerns the acoustic properties of the vowel stimuli. Specifically, the fundamental frequency (f0) of the vowel [o] was higher (by approximately 40–60 Hz) than those of the other vowels. This discrepancy was an inherent characteristic of the specific sound stimuli employed in the experiments. Since variations in f0 can potentially affect auditory processing, we cannot rule out the possibility that this elevated f0 influenced the participants’ key-press responses for [o]. Furthermore, the highest rejection rate after cutoffs was observed for [o] in Experiment 2 (17.91%). This higher rate of excluded trials could also be attributed to the elevated f0 of the [o] stimulus, further highlighting the potential influence of this acoustic difference on participants’ performance. Future research should utilize stimuli with more strictly controlled f0 values across all vowel categories to control for this potential confounding variable.

In conclusion, we conducted two behavioral experiments to verify whether spoken front vowels in Japanese promote key-press responses. In the response task of Experiment 1, the participants only pressed a key when they heard each spoken Japanese vowel. The results of Experiment 1 showed no significant differences in the response times to vowels. In the matching task of Experiment 2, the participants selected a pseudorandomly presented key corresponding to each spoken vowel. In Experiment 2, the results showed that the response times to the front vowels [i] and [e] were significantly faster than those to the back vowels [ɯ] and [o]. Essentially, this study argues that the auditory perception of front vowels could symbolically boost finger actions when participants consciously match spoken vowels with represented vowels.

## Data Availability

The authors uploaded the analyzed data to the Open Science Framework (https://doi.org/10.17605/OSF.IO/62NX9).

## Code availability

The codes used in this study are available on the Open Science Framework (https://doi.org/10.17605/OSF.IO/62NX9). The R codes and results for the performance data on the Open Science Framework are dated after the acceptance of this paper because the analysis for Experiment 2 was revised postacceptance, within a scope that does not change the paper’s conclusions.
